# Enhancing Electrochemical Biosensor Performance for 17β-Estradiol Determination with Short Split—Aptamers

**DOI:** 10.3390/bios12121077

**Published:** 2022-11-25

**Authors:** Normazida Rozi, Sharina Abu Hanifah, Nurul Huda Abd Karim, Lee Yook Heng, Sayuri L. Higashi, Masato Ikeda

**Affiliations:** 1 Department of Chemical Sciences, Faculty of Science and Technology, Universiti Kebangsaan Malaysia, Bangi 43600, Selangor, Malaysia; 2 Polymer Research Centre, Faculty of Science and Technology, Universiti Kebangsaan Malaysia, Bangi 43600, Selangor, Malaysia; 3 Department of Chemistry and Biomolecular Science, Faculty of Engineering, Gifu University, 1-1 Yanagido, Gifu 501-1193, Japan

**Keywords:** aptamer, split-type biosensor, polymer-based sensor, environmental monitoring, endocrine disrupting chemicals

## Abstract

Chronic exposure of 17β-estradiol (E2) even at low concentration can disorganize the endocrine system and lead to undesirable health problems in the long run. An electrochemical biosensor for rapid detection of E2 in water samples was successfully developed. The biosensor was based on split DNA aptamers attached onto poly (methacrylic acid-*co*-n butyl acrylate-succinimide) microspheres deposited on polypyrrole nanowires coated electrode (PPY/PMAA-NBA). The sandwich paired of split DNA aptamers used were truncated from 75 mer parent aptamers. These two strands of 12-mer and 14-mer split DNA aptamers were then immobilized on the PMAA-NBA microspheres. In the presence of E2, the split DNA aptamers formed an apt12-E2-apt14 complex, where the binding reaction on the electrode surface led to the detection of E2 by differential pulse voltammetry using ferrocyanide as a redox indicator. Under optimum conditions, the aptasensor detected E2 concentrations in the range of 1 × 10^−4^ M to 1 × 10^−12^ M (R^2^ = 0.9772) with a detection limit of 4.8 × 10^−13^ M. E2, which were successfully measured in a real sample with 97–104% recovery and showed a good correlation (R^2^ = 0.9999) with the established method, such as high-performance liquid chromatography. Interactions between short and sandwich-type aptamers (split aptamers) demonstrated improvement in aptasensor performance, especially the selectivity towards several potential interferents.

## 1. Introduction

The endocrine disrupting chemicals (EDCs) have become one of the main topics of research in the field of environmental science. An unpredictable exposure of EDCs from the environment has worrying impacts on human health, as they are capable to interfere with the function of the endocrine system, although in very low concentration. In a worst case, an incomplete removal of EDCs from conventional wastewater treatment plants (WWTP) has caused continuous discharged into the aquatic environment. The effluent from conventional WWTP still contain EDCs at range of concentration from ng/L to μg/L [1].

Among a wide range of compounds that have exhibited endocrine disrupting properties, the Environmental Protection Agency (EPA) has classified that 17β-estradiol (E2) in the lists of contaminants that required monitoring in public water from 2013 to 2015. In accordance with the Unregulated Contaminant Monitoring Rule 3 (UCRM 3), 17β-estradiol also listed by EU in a watch list of emerging pollutants in 2013 (Directive 2013/39/EU) [2] and EU Commission Implementing Decision 2015/495 [3] with minimum acceptable concentration of its 0.4 ng/L [4]. Two major sources that contribute the E2 into surface waters are runoff from field fertilized with manure and discharged into streams [4,5]. It is suspected to be a potent substance for female reproductive system as it has the strongest estrogen effect [5]. It is also known to interfere with normal endocrine function, further causing adverse effects on growth (premature puberty), metabolism and reproduction of the organisms and increasing the incidence of cancer (ovarian or breast) and tumor [6,7].

Enzyme-linked immunosorbent assay (ELISA) [8], high performance liquid chromatography (HPLC) [9], liquid chromatography and gas chromatography-mass spectrophotometer (GC/MS) [10,11] are the conventional methods for E2 determination [12]. Most of these conventional methods can provide an accurate and reliable results in detection of E2, but they require cumbersome and sophisticated instruments, complex pre-treatments, technical expertise and time consuming, which limits their usage for real time and in situ applications for environmental monitoring [13].

In recent years, there has been intensive research to detect the E2 compound using electrochemical methods based on various types of advanced materials modified electrode, such as poly(3,4-ethylenedioxylthiophene) [14], gold nanoparticles [15], carbon nanotubes [13], graphene [7] and carbon nanodots [16]. Among these electrochemical sensing methods, aptamer-based sensors (aptasensor) has gained popularity as aptamer based sensors could provide improved selectivity towards target compound [17,18,19].

Aptamer is typically single-stranded oligonucleotides screened from random-sequences DNA or RNA libraries using systematic evolution of ligands by exponential enrichment (SELEX) [20]. It can be easily synthesized and modified with other compound or functional group compared with antibodies. Unlikely, antibodies cannot be produced against toxic materials, while aptamer can be used to detect almost any molecule including molecule with high toxicity [21]. Despite of the availability of α-estrogen receptor antibodies to be used as recognition elements of E2, DNA aptamer is a better choice as it can bind specifically to their targets in the same manner as antibodies. This is because aptamer has higher affinity for their target [22] than those of antibodies. It also can withstand heat and extreme conditions. DNA aptamer will change their conformation to the hairpin loops shaped when interact with the target compound [23]. Consequently, aptamers have been widely used as recognition elements in aptamer-based biosensor.

However, most long sequence aptamers showed poor performance, such as 76-mer and 75-mer aptamers. As we know, excessive aptamers mean less sensitivity of competitive interaction dynamic among aptamer and target. Long aptamers tend to encapsulate the surface of nanostructures in the process, thus limiting the number of molecules able to bind on the nanostructure surface [24]. Single aptamer-based biosensors also tend to give lower sensitivity [25]. As for Liu et al. (2014) [26], they have split the 76-mer of E2 aptamer into a pair DNA aptamer to improve the sensitivity of the colorimetric sensor. This is because aptamer–target interaction occurs in a specific binding domain that might be only a fraction of the aptamer sequence for small molecule targets.

Nameghi et al. (2019) used a similar pair of DNA aptamers to develop E2 electrochemical aptasensor. Moreover, Alsager et al. (2015) [27] have hypothesized that eliminating excess bases will result in enhanced sensitivity of sensor. So, to improve the sensitivity of E2 biosensor, sandwich-type biosensor using a pair of short core sequences of DNA aptamer with a higher affinity to target can be applied. These aptamer’s pairs usually bind the same target at different sites.

Sandwich-type of aptamer based biosensors have been developed using various detection methods, such as electrochemical, surface plasmon resonance (SPR), optical and colorimetric platforms [25]. However, for E2 determination, sandwich-type of biosensor using a pair of aptamers has rarely been reported because of the non-availability of dual DNA aptamers. The emergence of advanced micro/nanomaterials and DNA aptamer capabilities has opened a new platform for the development an ultrasensitive aptasensor of E2. High surface to volume ratios characteristic of nanomaterials undoubtedly gained the most attention as it can lead to a higher sensitivity of aptasensor towards detection of target compounds [28]. Among different materials, conducting polymers (CPs), such as polypyrrole (PPY) polyaniline and polythiophene, have been widely studied.

The outstanding capability of CPs working at room temperature and their high conductivity would be main advantage for sensor application. Among known CPs, PPY has received great attention, not only due to its high conductivity and environmental stability, but also biocompatibility with bioreagents [29]. The conjugated π electrons (a system having coupled C=C bonds) in PPY contributed to the high electron affinity and can serve as electron mediators to improve the flow of electron in the redox reaction of [Fe(CN)_6_]^−3/−4^ on the surface of a working electrode [30]. PPY in various kind of nanostructures can be easily synthesized via chemical, physical and electrochemical methods [29,31,32]. In various applications, PPY offers high sensitivity in detection of a wide range of target compounds, such as nitrate [33], gases [28], arowana fish gender classification [34] and glucose [29,35].

A new approach enabling an electrochemical detection of 17β-estradiol (E2) in water sample using split DNA aptamer immobilized on the conductive polypyrrole/poly(methacrylic acid-co-n butyl acrylate) (PPY/P(MAA-nBA) has been proposed for simple, rapid and ultrasensitive detection of E2 in this study. This is aimed to improve the sensitivity of the E2 biosensor with shortened DNA sequences of aptamers splitting into two fragments to bind E2. PPY nanowires was also incorporated to enhance the response of the electrode when E2 aptamer binding event occurs.

## 2. Materials and Methods

### 2.1. Chemicals and Reagents

All reagents mentioned in this paper were used without further purification. The E2 split aptamers with the following sequences derived from Alsager et al. (2015) were synthesized by Macrogen (Malaysia): 5′-NH_2_-ATGCCGTTTGGG-3′ contains of 12-mer (apt12) and 5′-CCCAAGTTCGGCAT-NH_2_-3′ contains of 14-mer (apt14). Methacrylic acid (MAA), 17β-estradiol (E2), bovine serum albumin (BSA), bisphenol A (BPA), estriol (E3), progestrone (P4), potassium hexacyanoferrate trihydrate (II) (K_4_[Fe(CN)_6_]·3H_2_O), ethanol and potassium hexacyanoferrate trihydrate (III) (K_3_[Fe(CN)_6_]) were purchased from Acros Chemicals. The monomer of *n*-butyl acrylate (nBA), pyrrole, tris(hydroxymethyl)aminomethane (tris-HCl), photoinitiator 2-2-dimethoxy-2-phenylacetophenone (DMPP), ethylene glycol dimethacrylate (EGDMA), sodium dodecyl sulfate (SDS), methyl orange, iron(III) chloride hexahydrate (FeCl_3_·6H_2_O) and magnesium chloride (MgCl_2_) were purchased from Sigma-Aldrich. Sodium chloride (NaCl), hydrochloric acid (HCl) and potassium chloride (KCl) were purchased from Thermo Fisher. All aqueous solutions were prepared with deionized water (Sartorius, Germany) with resistivity no less than 18 MΩ cm.

### 2.2. Apparatus and Instrumentation

Cyclic voltammetry (CV) and different pulse voltammetry (DPV) were performed using a Dropsense potentiostat (Methrohm, Singapore). The 17β-Estradiol (E2) aptasensor was fabricated on the screen-printed electrode (SPE) (Scrint Technology (M) Sdn. Bhd) and was used as working electrode. A rod-shaped glassy carbon electrode and Ag/AgCl electrodes (containing 3.0 M KCl) were used as auxiliary and reference electrodes with scan rate of 0.05 mV/s, respectively. The potential used ranged from −1.0 V to 1.0 V was applied in all measurements. All homogeneous mixture of material solutions were prepared using sonicator bath Elma S30H. The morphology and chemical characterizations of nanowires and microspheres were measured using Scanning Electron Microscope (SEM) (ZEISS, Jena, Germany) and Fourier Transform Infrared (FTIR) (Perkin Elmer, Massachusetts, United State). The fluorescence spectra were measured using spectrofluorometer (Thermo Fisher Scientific Inc., Wilmington, NC, USA).

### 2.3. Preparation of Polypyrrole Nanowire

Polypyrrole (PPY) nanowire was synthesized as reported [36] (Li et al. 2017) with some modification. At first, 5 mM of pyrrole monomer and 0.25 mM methyl orange were dissolved in 50 mL deionized water. The mixture was sonicated for 2–3 min and manually shaken until all compounds were homogenously dissolved. Then, the solution was cooled in a refrigerator at 4 °C for 60 min and then 50 mL of pre-cooled aqueous solution iron (III) chloride hexahydrate (5 mM) was added dropwise into the solution. The molar ratio monomer to oxidant in the final solution was 1:1. The reaction solution were left undisturbed for 24 h to be completed when the precipitation of black PPY was observed. The products obtained were filtered and rinsed with 0.2 M hydrochloric acid, followed by ethanol and deionized water at least three times. The final product was dried overnight at a temperature of 40 °C. Scanning electron microscope (ZEUS, SEM) and Fourier Transform Infrared (FTIR) (Perkin Elmer, Massachusetts, United State) were conducted to characterize the nanowires.

### 2.4. Synthesis of Polymer Microspheres

Microspheres consisting of poly(methacrylic acid-*co*-n-butyl acrylate) (poly(MAA-*co*-nBA) were synthesized using photo-polymerization technique in the form of emulsion. A mixture of 90% *v*/*v* of methacrylic acid monomer (MAA), 10% *v*/*v* of n-butyl acrylate monomer (nBA), 0.09 g DMPP, 1 mg sodium dodecyl sulfate (SDS) and 3 mL deionized water was sonicated at room temperature for 15 min. The obtained milky solution was then photocured for 10 min with ultraviolet light under continuous nitrogen gas flow. Then, the microspheres were collected by centrifugation at 13,000 rpm for 30 min and carefully washed with Tris-HCl buffer (pH 7.5) three times and left to dry at ambient temperature. Scanning Electron Microscope (SEM) and Fourier Transform Infrared spectrometer (JEOL, FTIR) were used to evaluate the size of microspheres and the presence of functional groups in the microspheres, respectively.

### 2.5. Interaction Study between Split Aptamers and 17β-Estradiol (E2)

**Figure 1:**
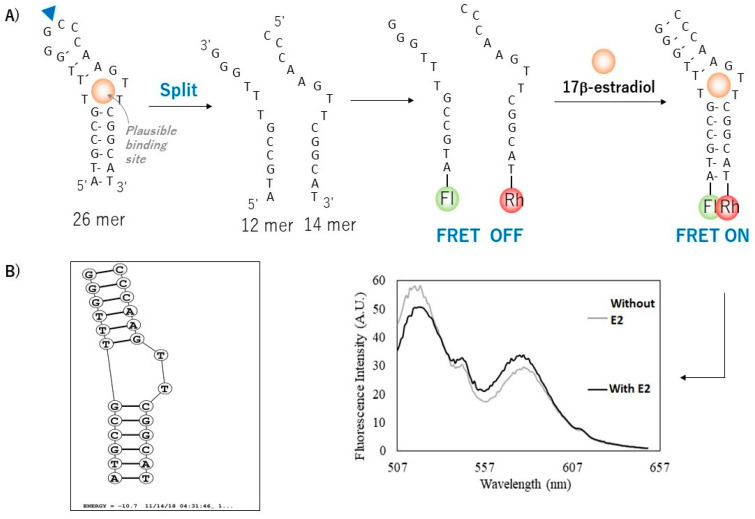
(**A**) split fragment of aptamer from 26-mer to the 12-mer and 14-mer of aptamer for FRET assay analysis. (**B**) Structure of 26-mer of aptamer fragment extract from software of RNA structure version 6.0.1.

Based on the 75-mer parent DNA aptamer reported by [27] Alsager et al. (2015), two short sequences of 12-mer and 14-mer paired aptamers were designed. As shown in Figure 1, these two short aptamers were modified with fluorescent probes, such as fluoroscein-5-isothiocyanate (FITC) and rhodamine isothiocyante (RITC) at the 5′ and 3′ ends; FITC was attached to the 5′ end of aptamer (apt12) and RITC was attached to the 3′ end of aptamer (apt14). These modified aptamers were synthesized and purified by Macrogen Company (Singapore) and used as received. The lyophilized powder was dissolved in a binding buffer (Tris-HCl buffer pH 7.5) and stored at 4 °C before use. The binding buffer (pH 7.5) was prepared by mixing 100 mM Tris-HCl, 200 mM NaCl, 25 mM KCl, 10 mM MgCl_2_ and 5% *v/v* ethanol. The concentrations of the both split aptamers were evaluated by measuring absorbance at 260 nm with a nanoDrop 2000 c Scan UV-vis spectrophotometer (Thermo Fisher Scientific Inc., Wilmington, NC, USA)). Fluorescence spectra to evaluate interaction between E2 and sandwich aptamers (apt 12 and 14) were recorded using spectrofluorometer at the excitation and emission wavelengths of 490 nm and 520 nm for FITC (12-mer aptamer) modified aptamer and 530 nm and 595 nm for RITC (14-mer aptamer) modified aptamer.

### 2.6. Fabrication of 17β-Estradiol (E2) Aptasensor

**Figure 2:**
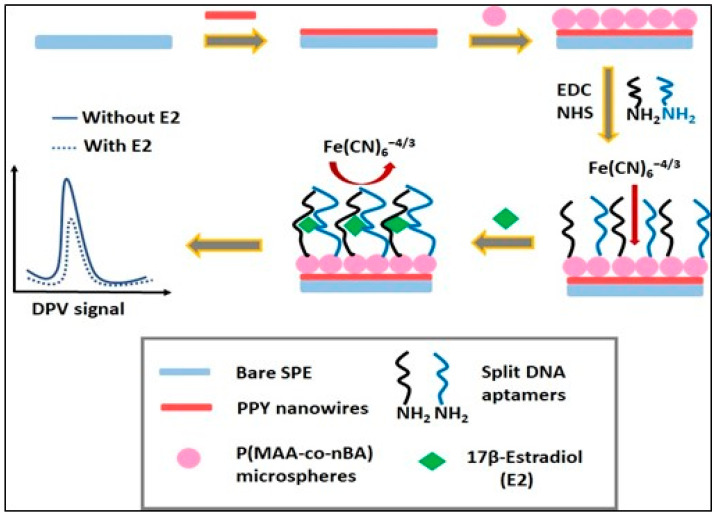
Schematic diagram to illustrate reaction of polypyrrole nanowire ((PPY/PMAA-nBA/APT) system.

Approximately 300 mg of microspheres were added in the 1 mL of EDC-NHS solution (0.01 M) and stirred for 24 h to activate the terminal carboxylic acid groups for aptamers immobilization. The fabrication of E2 aptasensor was begun with deposition of PPY nanowires (1 mg/100 µL) suspension in ethanol onto the carbon screen-printed electrodes (SPE) and left to dry at room temperature. Then, 10 µL of activated microspheres (2 mg/10 µL) were deposited on the electrodes and left to dry at room temperature. The procedure was continued by pipetting 10 µL containing apt12 (10 µM) and apt14 (5 µM) that were prediluted with Tris-HCl buffer (pH 7.5) was dropped onto the surface of modified electrodes to conduct a 12 h assembly reaction at room temperature. After 12 h, the electrodes were washed with Tris-HCl buffer solution (pH 7.5) to eliminate the unbound sandwich aptamers, then the electrode was incubated with 5 µL of E2 solution in different concentrations (1 pM to 1 µM), followed by washing with Tris-HCl buffer. Finally, differential pulse voltammetry (DPV) technique was tested to know if E2 can be detected DPV method with immobilized DNA split aptamers (Figure 2).

### 2.7. Optimization of 17β-Estradiol (E2) Aptasensor 

The split aptamer concentration was tested in range of 1 µM to 5 µM, whilst a pH value of binding buffer solution was optimized from pH 6.5 to 8.5 to get the best working conditions for the detection of E2. An optimum condition of buffer solution including aptamer binding buffer was important to maintain the physical stability of aptamer and its affinity towards target substances [37]. The optimization of the buffer solution including the pH effect and ionic strength were carried out to provide an optimum condition for the aptasensor to detect the E2 compound. The effect of incubation time between split aptamers and E2 compound on the current response also was evaluated in a range of 15 to 250 min. The response time of the aptasensor in the redox solution was also being measured in a range of 0 to 600 s. For each parameter, $30\times 10^{-9}$ M of E2 was used in the experiment and detected using DPV method.

### 2.8. Electrochemical Analysis of Aptasensor

An evaluation of the 17β-Estradiol (E2) aptasensor was studied in a series of E2 concentration in range of $1\times 10^{-4}$ to $1\times 10^{-12}$ M to obtain a linear response range and the detection limit of the aptasensor. This reaction involved 0.05 M of potassium hexacyanoferrate trihydrate (III) (K_3_[Fe(CN)_6_]), in pH 7.5 of Tris HCl buffer solution as a redox indicator. The differential pulse voltammetry (DPV) technique was used to measure the response with scan rate of 0.05 V/s and potential range from −0.2 V to 0.7 V.

### 2.9. Selectivity, Stability and Reproducibility

Few EDCs were identified as the closest chemical structures to E2 that may interfere E2 response. The chemicals were 17α-ethinylestradiol (EE2), estriol (E3), progesterone (P4), bovine serum albumin (BSA) and bisphenol A (BPA). The aptasensor membrane was incubated in $30\times 10^{-9}$ M of each one among four interferences. Current response was measured with the same method for E2 detection. Relative response was calculated via current differences before and after incubation of 30 nM E2 or other interferences on aptasensor surface. Furthermore, stability of E2 aptasensor was tested using 30 electrodes. Each electrode was stored in refrigerator at 4 °C after used. Response of aptasensors were checked periodically within 30 days of storage starting from the first day’s measurement at $30\times 10^{-9}$ M of E2. Finally, the reproducibility of aptasensor response was recorded for three times using duplication of five electrode sample of polypyrrole nanowire ((PPY/PMAA-nBA/APT) from the same batch.

### 2.10. Real Samples

Lake water was used as real samples for the validation of E2 aptasensor response. Prior to analysis, the samples were pre-treated to remove solid interference by filtration method. The treated samples were then spiked with E2 concentration range from $1\times 10^{-9}$ to $100\times 10^{-9}$ M [16]. Percent of recovery was calculated and sensor response were recorded using DPV and validated by HPLC. HPLC analysis was performed using Shimadzu LC-2010 C (Shimadzu, Kyoto Japan) with built in UV-visible detector. The C18 analytical column (250 mm × 3.0 mm) was used a mixture of acetonitrile, methanol and water in ratio of 23:24:53 (*v*/*v*) as mobile phase. The mobile phase was filtered through nylon membrane (0.45 µM) before use. The injection volume was 10 µL and mobile phase was isocratically pumped at a flow rate of 0.9 mL/min. The E2 compound was detected in wavelength of 225 nm at column temperature of 40 °C [38].

## 3. Results and Discussion

### 3.1. Characterization of Polypyrrole Nanowires

**Figure 3:**
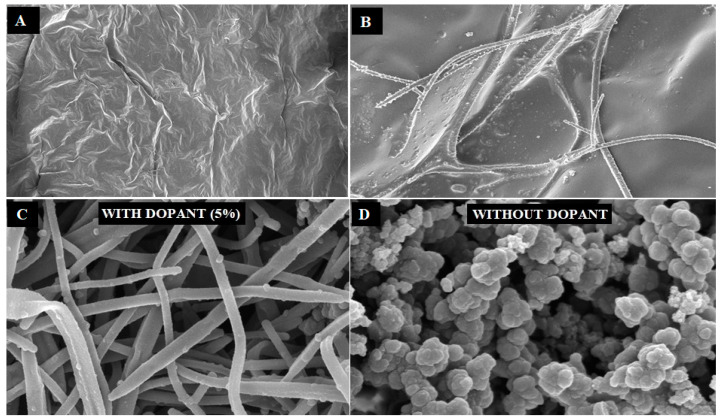
SEM images depicts on (**A**) methyl orange (MO) dopant formed as template for polymerization polypyrrole nanowires (PPY), (**B**) PPY nanowires started to polymerize on dopants, (**C**) PPY nanowires formed using MO as dopant and (**D**) PPY granule formed without dopant.

Figure S1 (Supplement Data) presents when a pre-cooled iron (III) chloride hexahydrate solution was added in a mixture of methyl orange and pyrrole, the solution started to change from an orange to a red color before it turned into a black solution after 3 to 4 h of reaction. It means that a process of formation of template for PPY growth when iron (III) chloride hexahydrate was added into mixture solution of PY monomer and methyl orange. The detail morphology of PPY nanowires was observed by scanning electron microscope (SEM). Formation of methyl orange (MO) dopant can be clearly seen in Figure 3A with soluble methyl orange salt converts to an insoluble acid under acidic conditions in a pH range of 3.2 to 4.4 [39]. As for Figure 3B, it depicts the process polymerization of PPY on MO dopants. The growth of PPY can be seen as started on the MO dopants. Figure 3C shows a micrograph that confirmed the formation of polypyrrole nanowires with the addition of methyl orange (MO) as dopants. Nanowires randomly entangled with diameter of 85–170 nm were found for a mixture of PPY-MO-FeCl_3_. In the absence of the methyl orange based template, reaction mixture of PPY-FeCl_3_ only formed globular structures of PPY (Figure 3D).

**Figure 4:**
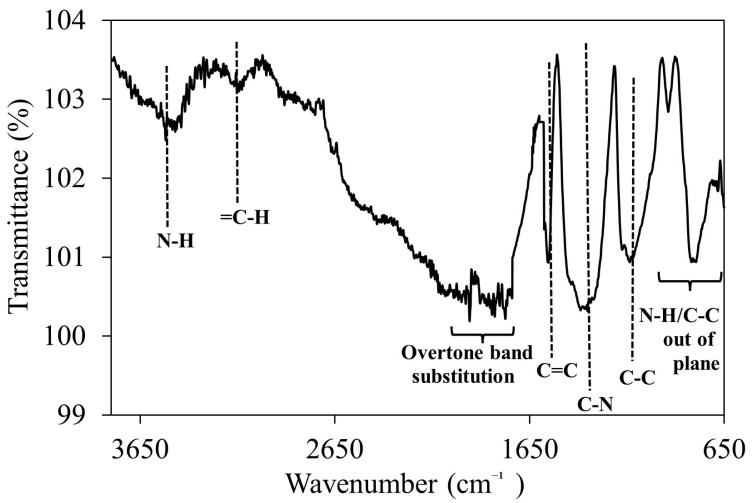
FTIR spectra of polypyrrole nanowires prepared in presence of MO as dopant.

Infrared spectra in the range of 650–3800 cm^−1^ were recorded for PPY nanowires (Figure 4). In the infrared spectra, the main bands of PPY were observed. The broad peak with maximum at 1556 cm^−1^ referred to the C=C stretching vibrations in the pyrrole ring [28,40,41]. The broad band center at 3450–3440 cm^−1^ due to the N-H stretching vibration of pyrrole units in polymers, which is compatible with the range of FTIR band reported by Zhao et al. (2016) [42] and Feng et al. (2013) [40]. The C-H stretching peak for *sp*^2^ appear at value greater than 3000 cm^−1^ [43]. In this case the C-H peak has reported approximately 3121 cm^−1^ for PPY nanowires. There is also peak at approximately 1741–2000 cm^−1^ corresponded to the weak overtone bands [43]. The peak corresponding to stretching vibration peak of C-N in PY rings of the PPY backbones was located at 1353 cm^−1^, which the stretch can occurs in the range 1000–1350 cm^−1^ [43,44]. In addition, a sharp peak at 1120 cm^−1^ exhibited C-H and N-H in plane deformation [45]. Lastly, peak of 938–793 cm^−1^ referred to C-C and N-H out of plane ring deformation are also present in the spectra [36]. Specifically, N-H out of plane bending absorption can sometimes be observed near 800 cm^−1^ [43]. The presence of these peaks confirms the synthesis of PPY.

### 3.2. Characterization of the Poly(methacrylic acid-co-n-butyl acrylate) (p(MAA-co-nBA)

**Figure 5:**
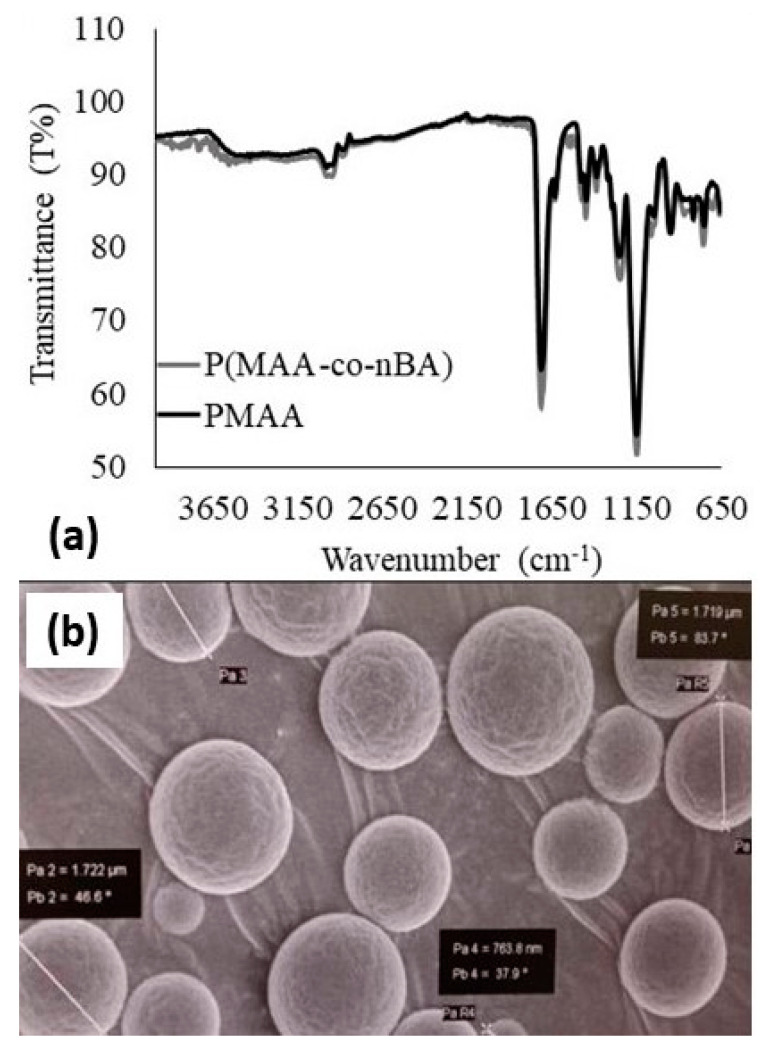
(**a**) FTIR spectra of p(MAA) and p(MAA-*co*-nBA) and (**b**) SEM images depict on p(MAA-*co*-nBA) microspheres with magnification of 20 k×. The 9:1 composition of PY:nBA monomers was used in this reaction.

Poly(MAA-*co*-nBA) microspheres were prepared from methacrylic acid (MAA) and *n*-butyl acrylate (nBA). It shows a broad -OH stretching vibration peak at 3504 cm^−1^ to 3030 cm^−1^ for p(MAA-*co*-NBA) and p(MAA) microspheres (Figure 5a). These peaks can be associated with methacrylic acid carboxylic group (-COOH). Lampman et al. (2010) [43] has stated that O-H group in carboxylic acid group would stretch, usually very broad (strongly H-bonded) and often overlap the C-H absorptions. Similarly, Harun et al. (2019) [46] and Belkadi et al. (2018) [47] have also reported a broad -OH peak around 3600 cm^−1^ to 3100 cm^−1^. Peaks at 2985, 2884 cm^−1^, 1449 cm^−1^, and 753 cm^−1^ were assigned to the aliphatic carbons of the polymer chain backbone [48] (Bajpai et al. 2009). The -CH_2_ and -CH_3_ stretching peak were observed at 2985 to 2884 cm^−1^ can be attributed for PMAA and copolymer. These peaks presented in the spectra due to the presence of sp^3^ hybridization -CH stretch formed in the structure. In addition, peak at 753 cm^−1^ was assigned for C-H_2_ bending (rocking) motion associated with four or more C-H_2_ group in a chain (long chain band). The C=O group presented in carboxylic acid group of MAA and ester group of nBA could be observed at 1715 cm^−1^ and 1631 cm^−1^ for both spectra. Apparently, there is no -C=C- group peak observed in both spectra, as usually the stretching peak can be identified near 1600 cm^−1^ with lowered frequency and increase in intensity [43,48]. So, completion of polymerization for both microspheres confirmed by the disappearance of C=C group in both spectra and has successfully being synthesized by using emulsion polymerization. Lastly, the signals at 1263 cm^−1^ to 1148 cm^−1^ indicated the presence of C-O group in the acrylate structure of nBA and carboxylic acid group of MAA [47]. Each peak in copolymers FTIR spectra (Figure 4a) shows an increase in transmittance intensity. It may happen to be due to the addition of 10% of nBA monomer incorporated in the copolymer. Figure 5b present morphology of p(MAA-*co*-nBA) and reveals the spherical shapes with diameter size from 0.763 µm to 1.722 µm.

### 3.3. Interaction Study between Sandwich Aptamers and 17β-Estradiol (E2)

The possible interaction of split aptamers with E2 in a sandwich format was investigated. Simply, the previously reported 75-mer aptamer sequence [27] was truncated and shortened into two short fragments of split aptamers (apt12 and apt14), which bear Forster Resonance Energy Transfer (FRET) paired fluorescence probes (FITC for apt12 and rhodamine for apt14, respectively).

**Figure 6:**
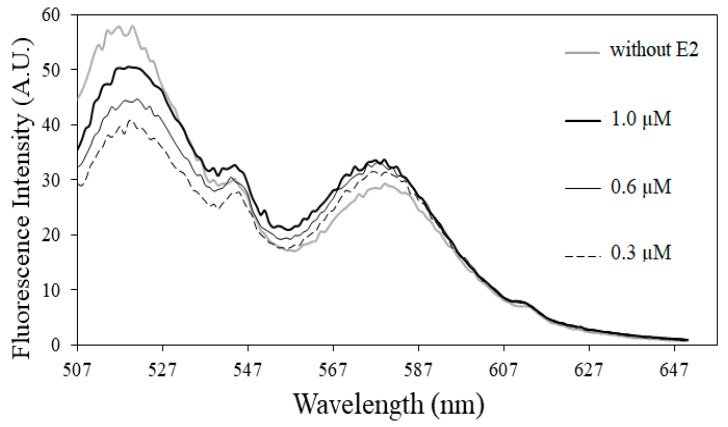
Emission spectra of split 12 and split 14 showing enhanced FRET by the addition of E2 in the concentration range from 0.3 to 1.0 µM, upon excitation at 490 nm.

Fluorescence spectral change was monitored to investigate the interaction between split aptamers and E2. Upon E2 binding, equilibrium of hybridization between sandwich aptamers should shift toward complex formation and simultaneous conformational change of the hybridized complex could also occur, which can be readout by a FRET-ratio change. As shown in Figure 6, upon excitation of FITC at 548 nm, the fluorescence intensity of rhodamine (FRET acceptor) increased while that of FITC (FRET donor) decreased by the addition of E2 in a concentration dependent manner. This enhanced FRET suggests the formation of apt12-E2-apt14 sandwich complex and a good binding behavior of the split aptamers with E2. Thus, the pair of split aptamers were used subsequently to develop an electrochemical biosensor for E2.

### 3.4. Electrochemical Properties of 17β-Estradiol (E2) Aptasensor

**Figure 7:**
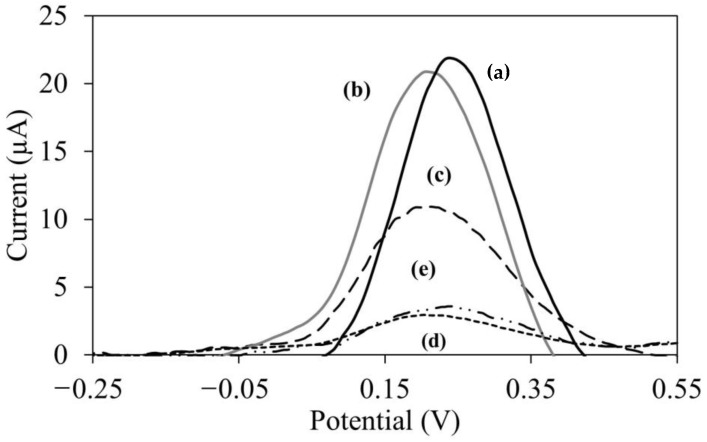
DPV profile of (a) Poly(methacrylic acid-co-n butyl acrylate)/Polypyrrole nanowires electrode (PPY/PMAA-nBA), (b) Poly(methacrylic acid-co-n butyl acrylate)/Polypyrrole nanowires/split aptamers electrode (PPY/PMAA-nBA/APT), (c) Modified electrode with an addition of 30 nM of E2 solution, (d) blank screen printed electrode (SPE) and (e) P(MAA-nBA) electrode only without PPY nanowires. The DPV parameter in range of −0.25 V to 0.55 V with scan rate of 0.05 V/s and 0.01 M of tris buffer at pH 7.5.

Figure 7 shows electrochemical properties of split aptamer. In the absence of PPY, a very low current reading was observed for unmodified screen-printed electrode (SPE). The deposition of PMAA-nBA onto the electrode surface also could not improve the current reading due to non-conducting property of this copolymer. The less conductive behavior of these electrode materials caused a weak electron transfer between the redox indicator [Fe(CN)_6_]^−3/−4^ and the electrode surface. In order to increase the conductivity of aptasensor electrode surface, PPY nanowires was added on top of the PMAA-nBA coated electrode.

Meanwhile, when E2 were present on the surface of PPY/PMAA-nBA/APT, the successful binding between two aptamers and E2 has also produced a split12-E2-split14 complex that became a physical barrier on the electrode surface, which significantly prohibited the access of [Fe(CN)_6_]^−3/−4^ to the electrode surface. This condition has led to a further diminished of electrochemical current since the apt12-E2-apt14 sandwich complex formed on the electrode surface hindered the redox reaction of [Fe(CN)_6_]^−3/−4^. In contrast, in the absence of E2, aptamers bridging process did not occur on the surface of the electrode and this resulted in easier access of the redox [Fe(CN)_6_]^−3/−4^ to the electrode surface. This yielded higher current value.

The PPY/PMAA-nBA modified screen printed carbon electrode (SPE) indicated the highest current recorded, due to the excellent electron transfer between the PPY/PMAA-nBA electrode and [Fe(CN)_6_]^−3/−4^. In contrast, PMAA-nBA modified electrode without PPY could not serve better electron transfer of redox reaction as the current recorded is very low. In this case, conductive polymer, such as PPY nanowire has proven to improve electrochemical property of the biosensors due to their unique features, such as have a large surface area and flexible for biomolecules immobilization [49]. These unique features have contributed to the improvement in the sensitivity of E2 aptasensor. However, when the sandwich aptamers (apt12 and apt14) were immobilized on the PMAA-NBA/PPY electrode, the current measured was slightly decrease which could cause by the electrostatic repulsion between phosphate skeleton of split aptamers and negatively charged of [Fe(CN)_6_]^−3/−4^ [12]. This repulsive effect hindered the approaching of redox agent onto the electrode surface.

### 3.5. Optimization and Evaluation of 17β-Estradiol (E2) Aptasensor

**Figure 8:**
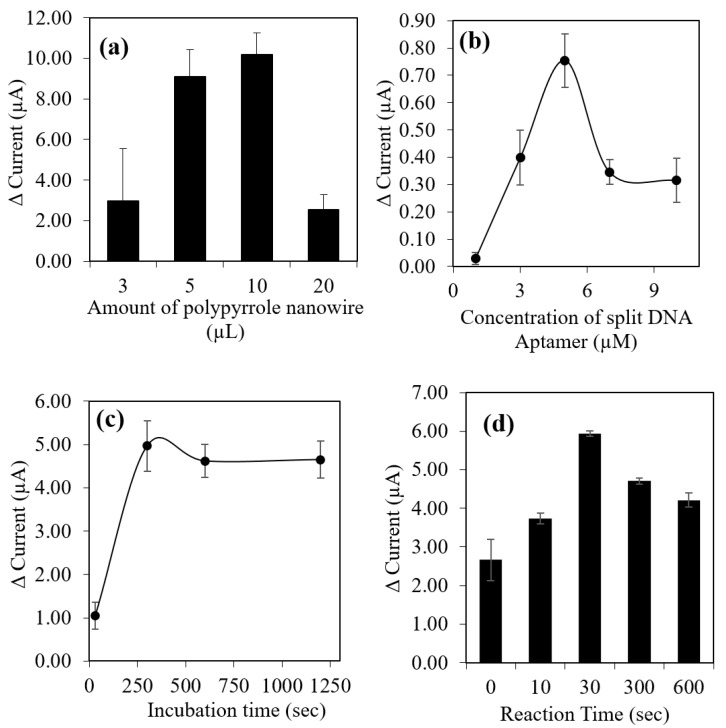
(**a**) Effect amount of polypyrrole nanowires, (**b**) effect concentration of split DNA aptamer, (**c**) effect of incubation time of E2 and (**d**) effect of reaction time. For each parameter, $30\times 10^{-9}$ M of E2 was used in the experiment and detected using DPV method. The scan rate of 0.05 V/s and potential between −1.0 V to 1.0 V was applied in the measurements.

In order to determine the optimum conditions for the E2 sensor response, few analyses were carried out including the amount of polypyrrole (PPY) nanowires, aptamer concentration, incubation time of E2 on the aptasensor and reaction time of aptasensor response. As shown in Figure 8a, an increasing amount of PPY in range of 3 µL to 10 µL has increased the relative current of electrochemical responses. The addition of PPY will contribute to the increased of conductivity of PPY and help in improving the electron transfer rate of E2 aptasensor [50]. However, when the amount of PPY increased more than 10 µL, the E2 aptasensor response declined due to the excess coverage of PPY nanowires on the surface of electrode and limit the electron transfer in the reaction [13]. The layer of PMAA-nBA/PPY started to peel off from the electrode surface as soon as it being incubated in [Fe(CN)_6_]^−3/−4^ solution, resulting in poorer response of E2 aptasensor. Furthermore, a fixed amount of 0.2 mg/µL of p(MAA-*co*-nBA) microspheres was coated on the PPY modified electrode, to prevent the aptasensor layer peeling off from the surface of the electrode and decreased the aptasensor responses. The p(MAA-nBA) microsphere was used as the matrix for the immobilization of the E2 split DNA aptamer.

The concentration effect of sandwich DNA aptamer on the modified electrode also need to be optimized. There was a drop in relative current response value after sandwich DNA aptamer was added of more than 5 µM shown in Figure 8b, due to high concentration of aptamer thus limit the aptamers’ elasticity. This type of aptasensor design only need minimum amount of aptamer to produce highly sensitive detection of E2. Therefore, optimum concentration of sandwich DNA aptamer at 5 µM was employed in the following aptasensor optimization experiments for optimal aptamer function.

For the incubation time effect of E2 compound on the aptasensor surface, Figure 8c has exhibited that relative current response increased progressively with increasing incubation times up to 4 min, indicating increased in amount of E2 compound bind to the DNA aptamer attached on the modified electrode. The relative current response slightly decreased and stabilized after 10 min of incubation time. The stabilization of relative response occurred due to the paired DNA aptamer assumed to be fully occupied with E2 compound to form apt12-E2-apt14 complex on the surface of aptasensor. On the other hand, Figure 8d shows the relative current response of aptasensor increased progressively with increasing of incubation time in [Fe(CN)_6_]^−3/−4^ solution from 0 to 30 s, which 30 s has exhibited highest relative current response toward detection of E2. However, the response started to decrease after 5 min. Therefore, an incubation time of 4 min and a response time of 30 s were selected as optimum condition for E2 aptasensor.

**Figure 9:**
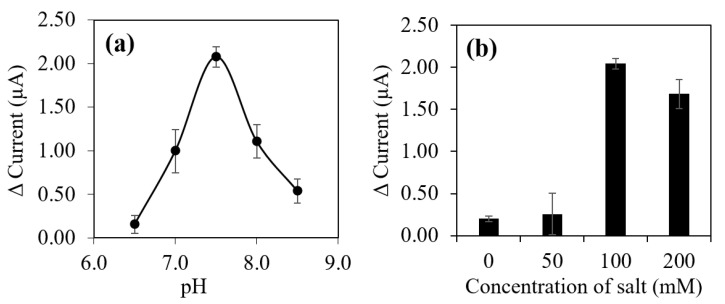
(**a**) Effect of pH and (**b**) effect of salt concentration on the aptasensor DPV response using $30\times 10^{-9}$ M as target analyte. The scan rate of 0.05 V/s and potential between −1 V to 1 V was applied in the measurements.

The most crucial aspect in optimization of the E2 aptasensor response was to ensure suitable buffer solution used in the reaction. Differing surrounding of pH values would give enormous impact on the degree of aptamer binding specificity as aptamer binds by complementary nucleic acid base pairing [51]. It will also cause either DNA activation or DNA destruction [52] (Chang and Yang 2012). The experiment results in Figure 9a suggested that in acidic conditions of tris buffer solution in pH 6.5, a lower relative current response was recorded due to the sugar phosphate backbone of nucleic acid was undergoing protonation reaction. Consequently, the protonation reaction has reduced the solubility of split aptamer molecules in aqueous surrounding and affecting configuration of immobilized split aptamers [53] as well as decreased the binding reaction rate between a pair of aptamer and E2 compound to form apt12-E2-apt14 complex. On the contrary, in alkaline pH of 8.0 to 8.5 the relative current response rapidly decreased due to the basic buffer solution has broken up weak hydrogen bond between nucleobases and resulted in denaturation of the entire immobilized sandwich aptamer [53].

This study indicated that the most favored pH for split DNA aptamer to form a physical apt12-E2-apt14 complex with E2 compound was pH 7.5 using tris buffer solution, which will be used in the subsequent study. Followed by the ionic strength was studied using tris buffer solution and Na^+^ ions. The pH concentration was influenced by buffer solution concentration and needed to be balanced with salt ion concentration to ensure the kinetic reaction and chemical equilibrium were maintained [54]. As shown in Figure 9b, at 50 mM concentration of Na^+^, lower relative current response was recorded as there was inadequate of Na^+^ ion in the reaction. This was ascribed to the presence of Na^+^ ions in tris buffer solution will facilitate in maintaining sandwich aptamer folded structure and allow E2 compound to bind with the immobilized sandwich DNA aptamer with high specificity. This fact can be confirmed when the concentration of Na^+^ ion increased up to 100 mM, a higher relative current response was measured. The optimum Na^+^ ion concentration in tris buffer solution was recorded at 100 mM, which will be used in the subsequent research.

### 3.6. Analytical Performance of 17β-Estradiol (E2) Aptasensor

**Figure 10:**
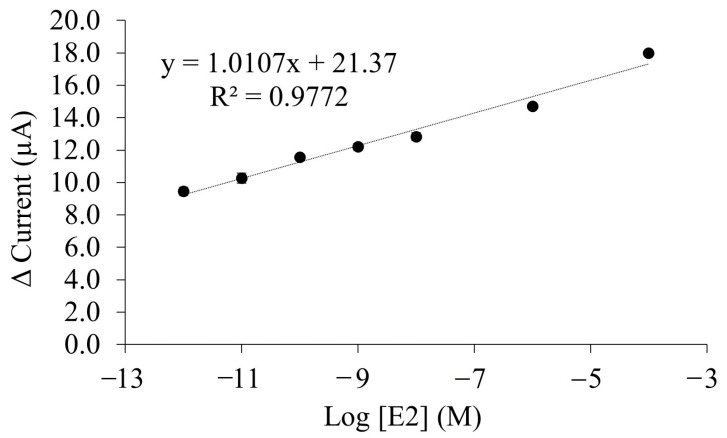
The calibration plot of linear relationship between relative peak current (I_0_-I) and the logarithm of E2 concentration (M). I_0_ and I are the current before and after addition of E2, respectively. The tris-buffer pH 7.5 with split aptamers concentration of 5 µM and reaction time of 30 s was applied in the experiment.

The performance of an analytical method was usually being investigated based on linear range and limit of detection (LOD) parameter. By employing DPV method under optimum conditions with potential range of −0.2 V to 0.9 V, the analytical performance of 17β-estradiol (E2) aptasensor was performed to investigate the relationship between the relative peak current and different concentration of E2. As showed in Figure 10, the highest relative peak current was observed at the lowest concentration of E2 (1 × 10^−12^ M), indicating that there was only least of apt12-E2-apt14 complexes were formed as physical barrier that passivate redox reaction of [Fe(CN)_6_]^−3/−4^ solution on the electrode surface. This condition has contributed to the highest relative peak was recorded. As E2 concentration increase, a decrease in peak current were observed in DPV voltammogram (Figure S2 in Supplement Data). Subsequently, Figure 10 exhibited that relative electrochemical signal is linearly proportional to the logarithm E2 concentration in range of 1 × 10^−4^ M to 1 × 10^−12^ M. The detection limit (LOD) of E2 aptasensor was determined at 0.48 pM (S/N = 3), which is lower than maximum residue of E2 in surface water (1.47 pM) determined by National Environmental Protection Agency of the United States [55]. The LOD value also was determined lower than Japan’s regulation of E2 limit in drinking water at 0.294 nM [55].

### 3.7. Specificity, Stability and Reproducibility

**Figure 11:**
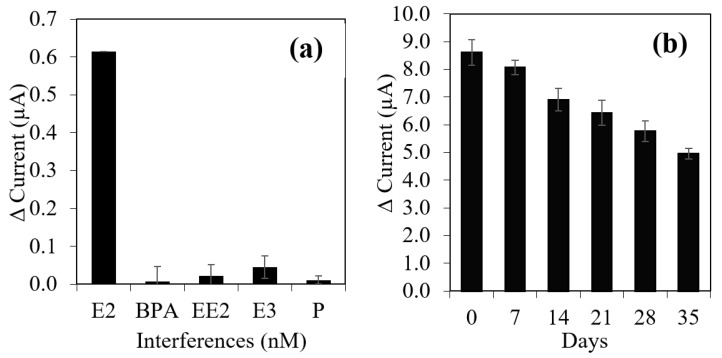
(**a**) Selectivity of the sensor: comparison of relative current response of the E2 aptasensor incubated with different types of interferences, the error bars were calculated based on three times measurement of each interferences ($30\times 10^{-9}$ M) with buffer solution pH 7.5. (**b**) The long-term storage stability test for E2 aptasensor in 35 days with storage temperature 4 °C.

To assess the specificity of the aptasensor, several endocrine disrupting chemicals compounds, such as 17α-ethinylestradiol (EE2), estriol (E3), progesterone (P) and bisphenol A (BPA) which have similar structures or coexists with E2 in environment were used as interferences. Relative current response caused by disrupting chemicals were calculated in Figure 11a. The results illustrated that only E2 led to a remarkable change of the relative current response of aptasensor in comparison with other interferents. The aptasensor only responded to E2 and there was no obvious influence toward detection of interferents via this aptasensor. This condition explained the high specificity of the proposed aptasensor, unaffected by the interferents and owned its specificity only towards E2 compound [56].

The long-term storage stability of the aptasensor was evaluated for over 35 days and was stored at 4 °C throughout the study. As shown in Figure 11b, the relative current response only maintained up to 94.6% than the original relative response in 7 days. These decrease in sensitivity were due to the inactivation of the binding aptamer or collapse of the aptasensor membrane [57]. The response decreased to 5.8% of original response in 7 days, suggesting that the aptasensor had good stability. Overall, the response kept on decreased up to 59.1% in 35 days. In order to inspect on the precision of the aptasensor, reproducibility would be one of the valid tools [56]. In this aptasensor, the reproducibility was assessed with relative standard deviation (RSD) of five electrodes each incubated in two different concentrations of E2, 1 × 10^−8^ M and 1 × 10^−9^ M. Five sample of similar aptasensor membrane that were fabricated in the same batch have recorded good reproducibility. Some factors, such as amount of polypyrrole (PPY) nanowires can cause a variation, however at the same time a good reproducibility recorded with RSD value of 1.48% and 1.55 % (*n* = 5), for both concentrations. It was probably due to amount of PPY was fixed for each electrode in effort to have a good reproducibility.

### 3.8. Real Samples

**Figure 12:**
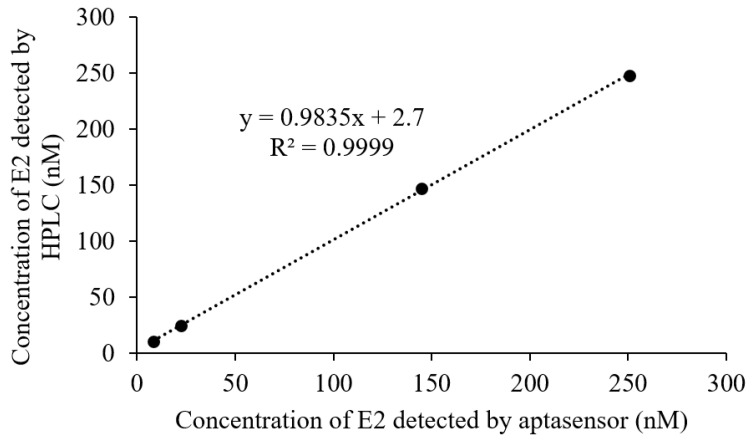
Correlation between E2 aptasensor and HPLC methods for determination of E2.

The application of aptasensor to detect E2 in water sample were tested to evaluate the feasibility of the developed method. As shows in Table 1, the recoveries results of the aptasensor in lake water were found within the range from 97.5% to 101.3% with RSD lower than 0.83% based on triplicate experiments at each concentration. It was indicated that the E2 aptasensor can be applied in detection of E2 in different environmental waters. Meanwhile, the concentration of E2 were validated using HPLC, in order to determine the reliability of the biosensor (Figure 12). The result of E2 determined by both methods have a good correlation between the two methods (Figure 12). There was good correlation between HPLC and aptasensor, suggesting that the aptasensor is promising for in situ detection.

### 3.9. A Comparison with Other Similar Aptasensors Reported

The advantages of the E2 sandwich type aptasensor developed here are apparent in the comparison with other E2 sensors shown in Table 2. For example, the electrochemical aptasensor reported here demonstrated better lower detection limit (LOD) than those reported based on antibody or enzyme and colorimetric transduction methods [26,27,30,58]. The LOD of aptasensor from this work is similar or slightly poorer than those reported using aptamer and electrochemical transduction [12,59,60]. However, when the selectivity of the aptasensors were examined, it was found that the aptasensor developed from split aptamers concept provided better selectivity. Thus the aptasensor from this work and that of Nameghi et al. 2019 [12] showed better selectivity when compared with that of Zhu et al. 2015 [59] and Na et al. 2016 [60], who used single and longer aptamers as recognition elements.

The observation that shorter split aptamers could provide better selectivity for an analyte is further confirmed here. The aptamers used in this work was 12- and 14-mer and the selectivity of E1 over E3 observed was 12 times whereas for Nameghi et al. [12] who used 33- and 43-mer aptamers for the same interferent was only 7 times. Thus, although the LOD and linear response range for the aptasensor developed here is similar to those reported but in terms of selectivity, it was much improved. The enhancement of the selectivity may be attributed to the use of a shorter split aptamer system.

This may be explained by an aptamer with short core sequence but higher affinity to target help to increase the ability to recognize of target [26]. This is because aptamer–target interaction occurs in a specific binding domain that might be only a fraction of the aptamer sequence for small molecule targets. Elimination of excess bases will result in enhanced selectivity of the sensor [27]. As compared to previous study that using shorter aptamer [27] and sandwich type aptamer [12,26], the use of 12 mer-14 mer couple in this work resulting in better performance in E2 aptasensor. Improvement of interaction between short and sandwich type aptamers in this study has attributed to a better selectivity. Thus, we can conclude that the improvement of aptasensor from this work (Table 1) could be attributed to factors, such as (i) an improvement in the electrode conduction and (ii) improvement in aptamer and E2 interaction after a truncation or split aptamer strands.

## 4. Conclusions

A split aptamer DNA aptasensor was successfully developed for E2 detection, based on a pair of short core sequences DNA aptamer and polypyrrole nanowire ((PPY/PMAA-nBA/APT) system. Polypyrrole coated onto SPE effectively improved the redox reaction of [Fe(CN)_6_]^−3/−4^ solution compared to unmodified electrode, thereby increasing the sensitivity of the detection method. Under optimized condition, the anodic peak current was linear in a E2 concentration range of 1 × 10^−4^ M to 1 × 10^−12^ M; the detection limit was 0.48 pM in tris-buffer solution pH 7.5. This aptasensor showed a good storage stability, which was retained up to 94.2% of its original response after 7 days of dry storage at 4 °C. The aptasensor designed here exhibited higher sensitivity, better selectivity, reproducibility, good recovery and rapid response (30 s) compared to longer split DNA aptamer in previous studies. The results of E2 analysis from the aptasensor produced were also correlated well with standard procedure for E2 analysis, such as the HPLC method.

## Figures and Tables

**Table 1:** Recovery of E2 from lake water samples (*n* = 3) using aptasensor.

E2 Concentration Added (nM) to Sample	E2 Concentration Found (nM)	Recovery (%)	RSD (% *n* = 3)
0.0	non-detected	-	-
1.0	0.99	99.0	0.65
5.0	4.89	97.7	0.62
10.0	10.13	101.3	0.45
25.0	24.63	98.5	0.59
50.0	48.78	97.5	0.43
75.0	73.51	98.0	0.42
100.0	101.00	101.0	0.83

**Table 2:** Previously reported E2 aptasensors and their analytical performances.

Sample	Detection Method	Linear Range (M)	LOD (M)	Ref.
AuNP/35-mer aptamer	Colorimetry	5.00 × 10^−8^ to 8.00 × 10^−7^	2.00 × 10^−9^	Alsager et al. 2015 [27]
AuNP/33-mer & 43-mer aptamer	Colorimetry	3.67 × 10^−10^ to 3.67 × 10^−4^	3.67 × 10^−10^	Liu et al. 2014 [26]
Polyclonal antibodies/Colloidal AuNP	Colorimetry	1.10 × 10^−11^ to 1.00 × 10^−7^	11 × 10^−12^	Minopoli et al. 2020 [58]
Poly 4,7-bis-(3,4-ethylenedioxylthiophene) thiophen-2-yl)benzothiadiazole/H_2_O_2_/Horseradish peroxidase enzyme	Electrochemistry (CV, DPV)	1.00 × 10^−7^ to 1.20 × 10^−4^	1 × 10^−6^	Spychalska et al. 2020 [30]
Poly(pyrrole-co-3-pyrrolacrylic acid)copolymer/75 aptamer	Electrochemistry (EIS)	1.00 × 10^−15^ to 1.00 × 10^−6^	1.00 × 10^−15^	Zhu et al. 2015 [59]
Ultrathin carboxylated polypyrrole nanotube/76 aptamer	Field effect transistor (FET) method	1.00 × 10^−15^ to 1.00 × 10^−6^	1.00 × 10^−15^	Na et al. 2016 [60]
Gold SPE/self-assembled 33-mer & 43-mer aptamer	Electrochemistry (DPV)	1.20 × 10^−12^ to 1.00 × 10^−10^ 7.00 × 10^−9^ to 1.00 × 10^−10^	5.00 × 10^−13^	Nameghi et al. 2019 [12]
PPY/PMAA-NBA/12-mer & 14mer aptamer	Electrochemistry (DPV)	1.00 × 10^−4^ to 1.00 × 10^−12^	4.80 × 10^−13^	This work

Note: Differential pulse voltammetry (DPV), Electrochemical impedance spectrometry (EIS), Cyclic voltammetry (CV).

## Data Availability

Not applicable.

## Supplementary Materials

The following supporting information can be downloaded at: https://www.mdpi.com/article/10.3390/bios12121077/s1, Figure S1: A mixture of 5 Mm iron (III) chloride hexahydrate, 0.25 mM methyl orange and 5 mM pyrrole monomer solution; Figure S2: DPV responses for E2 with different concentrations from a to g: 1 × 10-4 to 1 × 10-12 M, respectively. The tris-buffer pH 7.5 with split aptamers concentration of 5 μM and reaction time of 30 s was applied in the experiment.
