# Supplementary material for: Enhancing Electrochemical Biosensor Performance for 17β-Estradiol Determination with Short Split—Aptamers
Source: Biosensors (Basel). 2022 Nov 25;12(12):1077. doi: 10.3390/bios12121077 (PMC9776344; doi:10.3390/bios12121077)
Supplement: Supplementary file 1 [file biosensors-12-01077-s001.zip › biosensors-2036270-supplementary.pdf]

Supporting information

Enhancing Electrochemical Biosensor Performance for 17β-Estradiol Determination with Short Split—Aptamers

Normazida Rozi [1], Sharina Abu Hanifah [1,2,*], Nurul Huda Abd Karim [1], Lee Yook Heng [1], Sayuri L. Higashi [3] and Masato Ikeda [3]

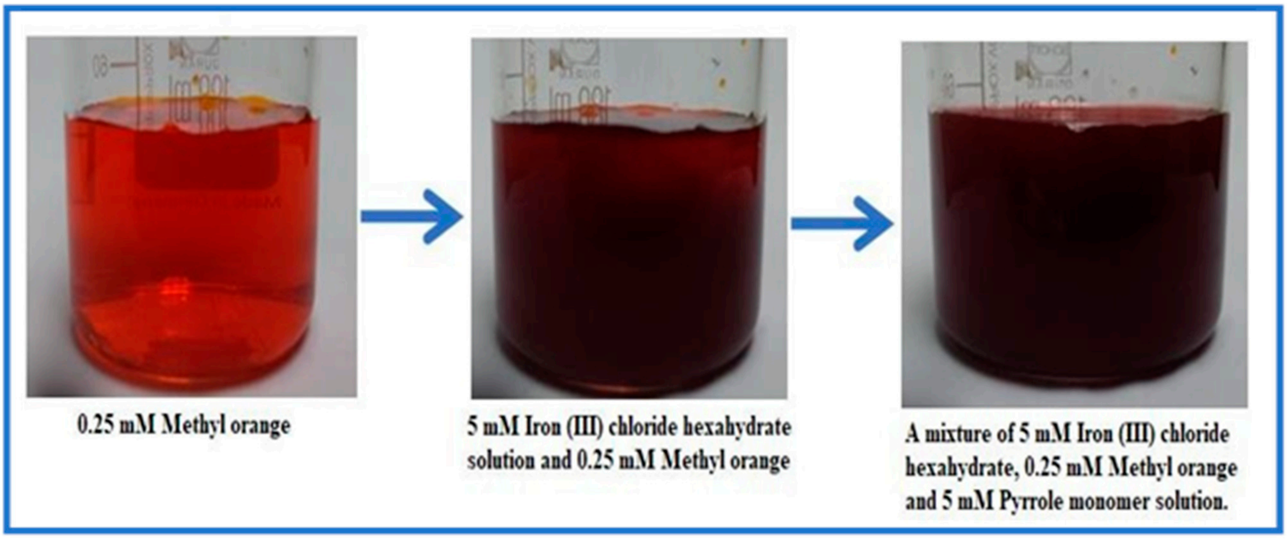

Figure S1. A mixture of 5 Mm iron (III) chloride hexahydrate, 0.25 mM methyl orange and 5 mM pyrrole monomer solution.

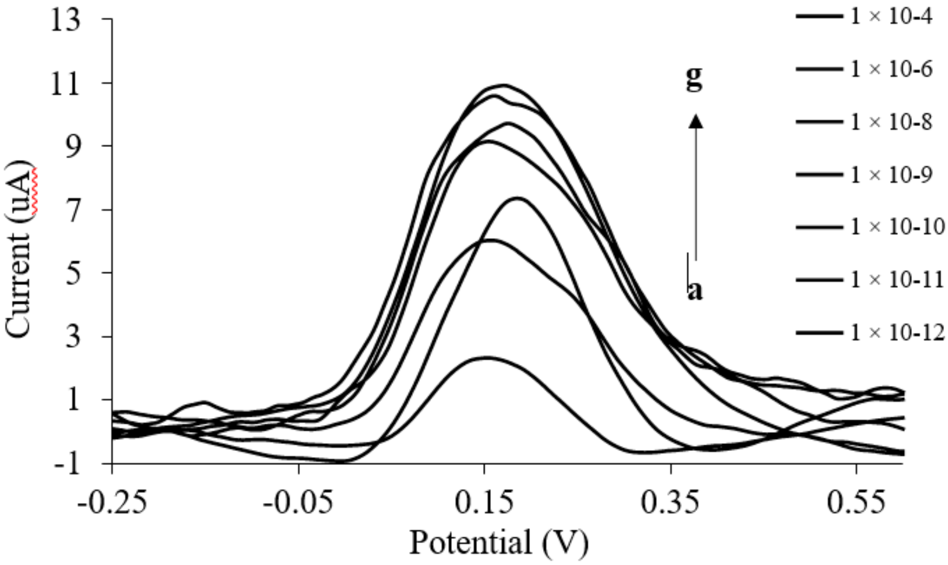

Figure S2. with different concentrations from a to g: 1×10^{-4} to 1×10^{-12} M, respectively. The tris-buffer pH 7.5 with split aptamers concentration of 5 μM and reaction time of 30 second was applied in the experiment.
